# Cultura de segurança do paciente entre profissionais de Enfermagem em um hospital filantrópico de Minas Gerais

**DOI:** 10.15649/cuidarte.1990

**Published:** 2021-09-10

**Authors:** Eduarda Vieira Magalhães, Fernanda Oliveira de Paiva, Maria Eduarda Soares Alves, Meire Cavalieri de Almeida

**Affiliations:** 1 Faculdade de Ciências Médicas e da Saúde de Juiz de Fora - Suprema. Juiz de Fora - MG, Brasil. E-mail: eduardavmag@gmail.com Faculdade de Ciências Médicas e da Saúde de Juiz de Fora Fora MG Brasil eduardavmag@gmail.com; 2 Faculdade de Ciências Médicas e da Saúde de Juiz de Fora - Suprema. Juiz de Fora - MG, Brasil. E-mail: fernandadepaiva16@gmail.com Faculdade de Ciências Médicas e da Saúde de Juiz de Fora Faculdade de Ciências Médicas e da Saúde de Juiz de Fora Juiz de Fora MG Brazil fernandadepaiva16@gmail.com; 3 Faculdade de Ciências Médicas e da Saúde de Juiz de Fora - Suprema. Juiz de Fora - MG, Brasil. E-mail: enf.mariaeduardasa@gmail.com Autor correspondente Faculdade de Ciências Médicas e da Saúde de Juiz de Fora Faculdade de Ciências Médicas e da Saúde de Juiz de Fora MG Brazil enf.mariaeduardasa@gmail.com; 4 Faculdade de Ciências Médicas e da Saúde de Juiz de Fora - Suprema. Juiz de Fora - MG, Brasil. E-mail: meirecavalieri@yahoo.com.br Faculdade de Ciências Médicas e da Saúde de Juiz de Fora Faculdade de Ciências Médicas e da Saúde de Juiz de Fora Juiz de Fora MG Brazil meirecavalieri@yahoo.com.br

**Keywords:** Segurança do Paciente, Cultura Organizacional;Avaliação em Enfermagem, Enfermagem., Patient Safety, Organizational Culture, Nursing Assessment, Nursing., Seguridad del Paciente, Cultura Organizacional, Evaluación en Enfermería, Enfermería.

## Abstract

**Introdução:**

Diferentes iniciativas têm sido realizadas com vistas à melhoria da qualidade do cuidado e segurança do paciente no Brasil. Contudo, o cumprimento dessas normas como uma série de requisitos parece não corresponder à real incorporação de mudanças e melhoria na segurança.

**Objetivos:**

Avaliar a cultura de segurança do paciente em um hospital filantrópico de Minas Gerais, observando, ainda, possíveis diferenças por turnos de trabalho, diurno e noturno.

**Métodos:**

Trata-se de um estudo observacional do tipo transversal. A população de estudo foi constituída por profissionais de Enfermagem atuantes nessa instituição filantrópica. Para a avaliação da cultura de segurança foi utilizado o Inquérito Hospitalar sobre Cultura de Segurança do Paciente (HSOPSC), criado pela *Agency for Healthcare Research and Quality* (AHRQ), validado, traduzido e adaptado transculturalmente no Brasil. Na análise e interpretação dos dados foram seguidas as orientações da AHRQ, sendo observadas dimensões fortalecidas e fragilizadas. Este trabalho foi aprovado pelo Comitê de Ética em Pesquisa.

**Resultados:**

Foram incluídos 118 participantes no estudo, sendo 17,2% desse total enfermeiros e o restante técnicos de Enfermagem. Nenhuma dimensão da cultura de segurança foi identificada como fortalecida na instituição. Foram consideradas como áreas frágeis: a “abertura à comunicação”, o “trabalho em equipe entre as unidades hospitalares”, a “adequação de pessoal”, as “mudanças de turno e transição entre as unidades”, e as “respostas não punitivas aos erros”. Em algumas dimensões foram observadas diferenças por turno de trabalho, diurno e noturno.

**Conclusão:**

A análise da cultura de segurança nesse hospital pode contribuir para um melhor direcionamento de condutas com vistas a garantir uma assistência mais segura.

## Abstract (en)

**Introduction:**

Different initiatives have been carried out with a view to improving the quality of patient care and safety in Brazil. However, compliance with these standards as a number of requirements does not seem to correspond to the actual incorporation of changes and improvement in security.

**Objectives:**

Evaluate the patient’s safety culture in a philanthropic hospital in Minas Gerais, also possible differences between work, day and night shiftes

**Methods:**

This is an observational cross-sectional study. The study population consisted of nursing professionals working in this philanthropic institution. For the evaluation of the safety culture, the Hospital Survey on Patient Safety Culture (HSOPSC), created by the Agency for Healthcare Research and Quality (AHRQ), validated, translated and adapted transculturally in Brazil was used. In the analysis and interpretation of the data, the guidelines of the AHRQ were followed, and strengthened and fragile dimensions were observed. This work was approved by the Research Ethics Committee.

**Results:**

We included 118 participants in the study, 17.2% of these total nurses and the rest of the nursing technicians. No dimension of the security culture has been identified as strengthened in the institution. Fragile areas were considered: “communication openness”, the “teamwork within units”, the “staffing”, the “handoffs and transitions”, and the “nonpunitive response to error”. In some dimensions, differences were observed by work shift, day and night. Conclusion: The analysis of the safety culture in this hospital can contribute to a better direction of conduct so as to ensure safer care.

## Resumen

**Introducción:**

Han sido llevadas a cabo diferentes iniciativas con el fin de mejorar la calidad del cuidado y la seguridad del paciente en Brasil. Sin embargo, el cumplimiento de esas normas como una serie de requisitos parece no corresponder a la real incorporación de cambios y mejora en la seguridad.

**Objetivos:**

Evaluar la cultura de seguridad del paciente en un hospital filantrópico de Minas Gerais, observando, además, posibles diferencias por turnos de trabajo, diurno y nocturno.

**Métodos:**

Se trata de un estudio observacional del tipo transversal. La población estudiada fue constituida por profesionales de Enfermería actuantes en esa institución filantrópica. Para la evaluación de la cultura de seguridad fue utilizado el Cuestionario Hospitalario sobre Cultura de Seguridad del Paciente (HSOPSC), creado por la *Agency for Healthcare Research and Quality* (AHRQ), validado, traducido y adaptado transculturalmente en Brasil. En el análisis e interpretación de los datos fueron seguidas las orientaciones de la AHRQ, observándose dimensiones fortalecidas y debilitadas. Este trabajo fue aprobado por el Comité de Ética en Investigación.

**Resultados:**

Fueron incluidos 118 participantes en el estudio, siendo 17,2% de ese total enfermeros y los demás técnicos de Enfermería. Ninguna dimensión de la cultura de seguridad fue identificada como fortalecida en la institución. Fueron consideradas como áreas débiles: la “apertura a la comunicación”, el “trabajo en equipo entre las unidades hospitalarias”, la “adecuación de personal”, los “cambios de turno y transición entre las unidades”, y las “respuestas no punitivas a los errores”. En algunas dimensiones fueron observadas diferencias por turno de trabajo, diurno y nocturno.

**Conclusión:**

El análisis de la cultura de seguridad en ese hospital puede contribuir para un mejor direccionamiento de conductas con el fin de garantizar una asistencia más segura.

## Introdução

A preocupação com a qualidade da assistência nos serviços de saúde é primordial, sendo os impactos positivos de um cuidado organizado e seguro, perceptíveis desde a atuação de Florence Nightingale no século XIX, quando foram observadas diminuições expressivas na mortalidade dos pacientes a partir de uma atuação mais sistematizada(1)-(2). Contudo, o tema da qualidade, que inclui a segurança do paciente, veio à tona de forma mais marcante somente em período recente, no fim do século XX, com a publicação de resultados alarmantes que estimaram entre 44.000 e 98.000 mortes anuais nos Estados Unidos devido a falhas de assistência à saúde(2)-(3).

No Brasil, onde elevados índices de eventos adversos também são observados(4), foi publicada, em 2013, a Portaria MS/GM ( Ministério da saúde/ Governo de Minas) n° 529/2013(5), que instituiu o Programa Nacional de Segurança do Paciente (PNSP). O objetivo geral do Programa é contribuir para a qualificação do cuidado em saúde para que erros sejam evitados nos estabelecimentos de todo o território nacional. Para isso, desde então, os estabelecimentos de saúde devem contar com um Núcleo de Segurança do Paciente para que diferentes eixos, como o estímulo à prática assistencial segura e o envolvimento do cidadão na sua segurança, sejam alcançados. Desse modo, devem ser promovidas e apoiadas implementações de diferentes iniciativas voltadas à segurança do paciente nesses espaços.

Conforme previsto pelo Ministério da Saúde, a adoção dessas iniciativas, nos diferentes estabelecimentos, vem aumentando desde então(6). Contudo, o cumprimento dessas normas como uma série de requisitos está longe de corresponder à sua real aplicação e incorporação de preocupação e compromissos constantes com a segurança. Em revisão sistemática que observou a chamada cultura de segurança do paciente em 21 países, com publicações entre 2005 e 2016, foi revelada predominância de culturas organizacionais subdesenvolvidas a fracas(7), alertando para a importância de que esse aspecto seja levado em consideração, principalmente em países em desenvolvimento, como o Brasil.

A cultura de segurança é uma característica fundamental presente nas chamadas organizações de alta confiabilidade, onde há uma preocupação constante acerca da segurança dentro do cenário organizacional, mas questionável nas organizações de saúde, reconhecidamente complexas. Comunicações inadequadas, falsa crença relacionada à isenção de erros devido à formação árdua, falta de comprometimento coletivo e aprendizado organizacional, insuficiência de recursos materiais e jornadas excessivas de trabalho entre os profissionais são alguns dos fatores relacionados à complexidade dessas organizações e ao comprometimento da qualidade e segurança dentro desses espaços(8).

Por ser produto de valores, atitudes e percepções do comportamento individual de cada profissional e também da equipe, onde todos devem estar comprometidos em oferecer um cuidado seguro, a cultura de segurança é fundamental à efetivação de práticas realmente seguras(9). Por isso, sua avaliação é considerada um indicador estrutural básico para promover iniciativas que visam à redução de riscos e a ocorrência de eventos adversos, principalmente em hospitais. Essa avaliação, além de benefícios gerenciais à própria organização, possibilita reflexões e aprendizados para cenários semelhantes, em prol do melhor cuidado ofertado ao paciente(10).

Devido à recente implementação de medidas oficiais direcionadas à segurança do paciente no Brasil e, ainda, devido à complexidade inerente às organizações de saúde, é possível que a cultura de segurança esteja fraca nesses espaços. No entanto, além da cultura de segurança nas organizações ser fundamental por interferir no cuidado prestado ao paciente, sua mensuração pode impactar em importantes mudanças de conduta(9)-(11). Desse modo, o objetivo deste estudo é avaliar a cultura de segurança do paciente entre os profissionais de Enfermagem em um hospital filantrópico de Minas Gerais.

## Métodos

Trata-se de uma pesquisa observacional do tipo transversal, de caráter descritivo e exploratório.

### População do estudo

A população de estudo foi constituída por profissionais de enfermagem, de nível superior e médio (enfermeiros e técnicos de Enfermagem), atuantes em instituição hospitalar filantrópica de um município mineiro. A amostra dos participantes incluídos foi do tipo não probabilístico e ocorreu por conveniência.

### Local do estudo

O estudo foi realizado em um hospital geral que presta assistência a diversas especialidades, localizado no município de Juiz de Fora, MG. Este hospital é uma associação civil de fins beneficentes e não lucrativos e possui 290 leitos ativos dedicados ao Sistema Único de Saúde (SUS). É credenciado pelo Ministério da Saúde para atendimento em alta complexidade que teve inicio em 2012 onde passou a ser realizados procedimentos e atendimentos como Cirurgia Bariátrica, Ortopedia e Traumatologia, Oftalmologia, Cirurgia Cardíaca Adulta e Pediátrica e Cardiologia Intervencionista, e também realiza atendimento às gestantes de alto risco provenientes da sua macrorregião no estado. Além disso, é associado ao Centro Nacional de Regulação de Alta Complexidade para atender a crianças e adultos de todo o país para cirurgias cardíacas e procedimentos de Hemodinâmica. Desde de 2012 é reconhecido pela Organização Nacional de Acreditação (ONA) como hospital acreditado, título este que é reavaliado anualmente.

### Critérios de elegibilidade, inclusão e exclusão

Foram elegíveis a participar do estudo todos os profissionais de Enfermagem, de ambos os sexos, atuantes em setores onde o funcionamento é ininterrupto na instituição, ou seja, naqueles onde há profissionais atuantes durante as 24 horas do dia. Esses setores correspondem às Unidades de Terapia Intensiva (UTI) adulto e neonatal, enfermarias, Centro Cirúrgico (CC), Centro de Parto Normal (CPN) e Central de Material e Esterilização (CME). Como critérios de inclusão foi estabelecido que os profissionais deveriam ter sido admitidos no setor há mais de dois meses, estivessem em serviço no momento da coleta de dados e apresentassem interesse e disponibilidade de participar após explicitados os objetivos da pesquisa e assinado o Termo de Consentimento Livre e Esclarecido (TCLE).

Foram excluídos os indivíduos que não retornaram com o questionário no período de coleta de dados.

### Instrumentos e medidas

A coleta de dados ocorreu a partir do preenchimento, pelos próprios profissionais incluídos no estudo, de um questionário autoaplicável que contemplou variáveis sociodemográficas - para a caracterização da amostra - e questões voltadas à avaliação da cultura de segurança do paciente.

Para a avaliação da cultura de segurança foi utilizado o instrumento *Hospital Survey on Patient Safety Culture* (HSOPSC)(10). Traduzido como Inquérito Hospitalar sobre Cultura de Segurança do Paciente (HSOPSC), este instrumento foi criado pela *Agency for Heathcare Research and Quality (AHRQ)*, dos Estados Unidos, em 2004(12) e teve sua adaptação transcultural e validação no Brasil em 2012(10),(13). No instrumento há quarenta e duas questões relacionadas à cultura de segurança do paciente, as quais são agrupadas em doze dimensões, a saber: Trabalho em equipe dentro das unidades; Expectativas do supervisor/chefe e ações promotoras da segurança; Aprendiza- do organizacional - melhoria contínua; Apoio da gestão hospitalar para a segurança do pacien- te; Percepção geral da segurança do paciente; Retorno da informação e comunicação sobre os erros; Abertura da comunicação; Frequência da notificação de eventos de segurança; Trabalho em equipe entre as unidades hospitalares; Adequação de pessoal; Mudanças de turno e transi- ções entre unidades/serviços; Respostas não punitiva aos erros.

A avaliação de cada dimensão é realizada a partir do percentual de respostas positivas, neutras e negativas. Valores percentuais mais altos indicam atitudes positivas em relação à cultura de segurança(14).

### Procedimento de coleta de dados

A coleta de dados foi realizada após a aprovação do Comitê de Ética em Pesquisa (CEP), tendo ocorrido em setembro de 2019. Pesquisadoras treinadas abordaram os participantes elegíveis na saída dos turnos de trabalho, explicitando os objetivos do estudo, apresentando o TCLE e convidando-os a participar. Aos que aceitaram, foi fornecido o instrumento de coleta, sendo o plantão seguinte a data acordada entre pesquisadora e participante para seu recolhimento. Aos participantes também foi esclarecido que o tempo despendido para a participação, referente ao preenchimento do questionário, seria de aproximadamente quinze minutos.

### Análises estatísticas

A análise descritiva da amostra foi feita por meio de frequências para variáveis categóricas e medidas de tendência central para variáveis contínuas. Na análise e interpretação dos dados quanto à cultura de segurança do paciente foram seguidas as orientações da AHRQ(14). Para avaliação do percentual de respostas positivas, neutras e negativas dentro de cada dimensão foi observado o quociente entre o número de cada categoria de resposta nos diferentes itens da dimensão e o número total de respostas válidas a esses mesmos itens. Esse percentual permitiu identificar áreas fortes e frágeis.

Foram consideradas dimensões fortalecidas da cultura de segurança do paciente aquelas cujas respostas positivas foram maiores ou igual a 75%. De modo semelhante, dimensões de cultura fragilizada e que necessitam melhoria foram aquelas cujas respostas positivas representaram 50% ou menos das respostas. Foram incluídas como respostas positivas, as dos itens escritos positivamente com respostas positivas (“concordo totalmente” ou “concordo”), ou dos itens escritos negativamente com respostas negativas (“discordo totalmente” ou “discordo”)(14).

As análises foram realizadas com o apoio do programa *Statistical Package for the Social Sciences* (SPSS) ® versão 17.

### Aspectos éticos

A pesquisa foi desenvolvida a partir dos pressupostos da resolução nº 466, de 12 de dezembro de 2012, que aprova as diretrizes e normas regulamentadoras de pesquisas envolvendo seres humanos e determina que todo e qualquer projeto de pesquisa que envolva seres humanos, direta ou indiretamente, deve ser submetido à apreciação do Comitê de Ética em Pesquisa.

O anonimato dos participantes está sendo mantido em todas as fases do estudo, assim como a privacidade e o sigilo das informações observadas, de forma a resguardar e preservar seus direitos. Nos questionários preenchidos não foi incluído o nome do participante. Esses questionários, assim como os bancos de dados estruturados para as análises, foram armazenados pelas pesquisadoras em local seguro, com acesso restrito à equipe de pesquisa.

## Resultados

**Figura 1:**
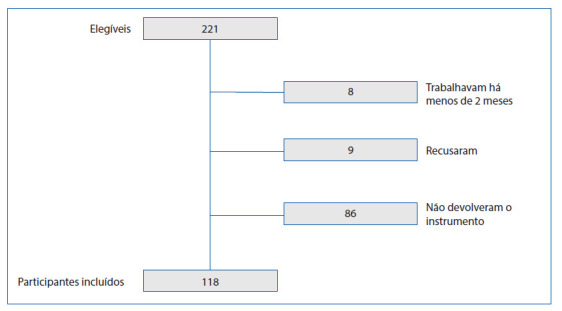
Fluxograma dos profissionais de Enfermagem elegíveis e incluídos no estudo Fonte:

Foram elegíveis a participar do estudo 221 profissionais de Enfermagem (enfermeiros e técnicos de Enfermagem) atuantes nos setores: Unidades de Terapia Intensiva (UTI) adulto e neonatal, enfermarias, Centro Cirúrgico (CC), Centro de Parto Normal (CPN) e Central de Material e Esterilização (CME). Dentre esses profissionais, foram excluídos: oito, por trabalharem na unidade há menos de dois meses; oitenta e seis, por não retornarem com o instrumento de coleta preenchido; e nove por recusarem-se a participar, totalizando 118 participantes incluídos no estudo. Na Figura 1 pode ser observado o fluxograma dos participantesincluídos.

Dentre os 118 profissionais incluídos, a maioria era do sexo feminino (82,7%), com idade entre 31 e 40 anos, apresentando em média 35 anos de idade. Quanto à formação, os técnicos de Enfermagem representaram 82,6% dos participantes e os enfermeiros, 17,2%. Esses profissionais relataram ter, em média, 6 anos de profissão, sendo que a maioria trabalha no hospital há menos de cinco anos (55,8%). Trabalham na instituição há menos de um ano, 10,6% dos profissionais incluídos; 23,9% tem tempo de trabalho no local entre 6 e 10 anos e 9,7% há mais de 11 e menos de 20 anos.

A maioria dos profissionais incluídos tem jornada de trabalho semanal na instituição maior ou igual a quarenta horas (75,2%) e exerce contato direto com os pacientes na sua área de atuação (94,0%). Houve uma distribuição homogênea dos participantes quanto ao setor de atividade e turno de trabalho, conforme pode ser observado na Tabela 1, juntamente com as demais variáveis sociodemográficas.

**Tabela 1:** Características sociodemográficas de 118 profissionais de Enfermagem incluídos no estudo

	N	(%)
Gênero			
Feminino	96/116	82,7
Masculino	20/116	17,3
Não informado	2/118	1,7
Idade (anos)	35 (mediana)	29 - 41,5 (IIQ)
20 - 30	36/113	31,9
31 - 40	46/113	40,7
41 - 50	25/113	22,1
≥ 51	6/113	5,3
Não informado	5/118	4,2
Grau de Instrução			
Ensino médio completo	75/114	65,8
Ensino superior incompleto	11/114	9,6
Ensino superior completo	10/114	8,8
Pós -graduação (especialização)	17/114	14,9
Pós -graduação (mestrado ou doutorado)	1/114	0,9
Não informado	4/118	3,4
Setores		
Diversas unidades/Nenhuma específica	12/118	10,2
Clínica (enfermaria)	35/118	29,7
Cirurgia (CC; CME)	14/118	11,9
Obstetrícia	14/118	11,9
Emergência	2/118	1,7
UTI (qualquer tipo)	41/118	34,7
Turno de trabalho		
Diurno	66/116	56,9
Noturno	50/116	43,1
Não informado	2/118	1,7
Cargo/Função			
Enfermeiro	20/116	17,2
Técnico de Enfermagem	96/116	82,8
Auxiliar de Enfermagem	-	-
Não informado	2/118	1,7
Tempo de profissão (anos)	6 (mediana)	3 - 10 (IIQ)
< 1 ano	2/106	1,9
1 - 5	50/106	47,2
6 - 10	34/106	32,1
11 - 20	16/106	15,1
≥ 21	4/106	3,8
Não informado	12/118	10,2
Tempo de trabalho no hospital (anos)		
< 1 ano	12/113	10,6
1 - 5	63/113	55,8
6 - 10	27/113	23,9
11 - 20	11/113	9,7
≥ 21	-	-
Não informado	5/118	4,2
Tempo de trabalho na unidade (anos)			
< 1 ano	21/114	18,4
1 - 5	65/114	57,0
6 - 10	20/114	17,5
11 - 20	7/114	6,2
≥ 21	1/114	0,9
Não informado	4/118	3,4
Tempo de trabalho semanal no hospital (horas)			
< 20	4/113	3,6
20 - 39	18/113	15,9
40 - 59	85/113	75,2
≥ 60	6/113	5,3
Não informado	5/118	4,2
Exerce contato direto com os pacientes			
Sim	109/116	94,0
Não	7/116	6,0
Não informado	2/118	1,7

*Legenda: IIQ (Intervalo Interquartil); CC (Centro Cirúrgico); CME (Central de Material e Esterilização); UTI (Unidade de Terapia Intensiva)*

Fonte: Elaboração própria a partir dos dados compilados no estudo

**Figura 2:**
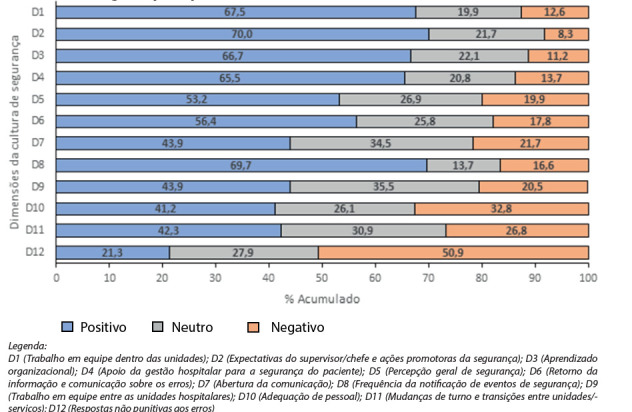
Frequência de respostas positivas, neutras e negativas para as doze dimensões da cultura de segurança do paciente

Na observação das respostas positivas, neutras e negativas, dadas pelos participantes, não foi identificada nenhuma dimensão da cultura de segurança fortalecida. Apesar disso, as dimensões quanto à “expectativa do supervisor e ações promotoras da segurança”,“frequência de notificação de eventos de segurança”, “trabalho em equipe dentro das unidades”, “aprendizado organizacional - melhoria contínua” e “apoio da gestão hospitalar para a segurança do paciente”, foram as que mais se aproximaram de uma cultura mais forte, com índices de respostas positivas acima de 65%, como observado na Figura 2.

Ao contrário, foram consideradas áreas frágeis e que requerem melhoria por terem respostas positivas menores que 50%: a “abertura à comunicação”, o “trabalho em equipe entre as unidades hospitalares”, a “adequação de pessoal”, as “mudanças de turno e transição entre as unidades”, e as “respostas não punitivas aos erros”.

*Legenda:*

*D1 (Trabalho em equipe dentro das unidades); D2 (Expectativas do supervisor/chefe e ações promotoras da segurança); D3 (Aprendizado organizacional); D4 (Apoio da gestão hospitalar para a segurança do paciente); D5 (Percepção geral de segurança); D6 (Retorno da informação e comunicação sobre os erros); D7 (Abertura da comunicação); D8 (Frequência da notificação de eventos de segurança); D9 (Trabalho em equipe entre as unidades hospitalares); D10 (Adequação de pessoal); D11 (Mudanças de turno e transições entre unidades/- serviços); D12 (Respostas não punitivas aos erros)*

Fonte: Elaboração própria a partir dos dados compilados no estudo

Ao serem categorizadas as respostas para avaliação das dimensões de cultura de segurança por turno de trabalho, foram observadas diferenças maiores entre os turnos diurno e noturno para: “aprendizado organizacional - melhoria contínua”, “retorno da informação e comunicação sobre os erros”, “frequência da notificação de eventos de segurança” e “adequação de pessoal”.

Ao ser observada isoladamente no período diurno a “frequência da notificação de eventos de segurança”, essa pode ser considerada uma área fortalecida da cultura de segurança (77,2%). No entanto, essa área alcançou somente 60,7% das respostas positivas dentre os profissionais do período noturno. O “aprendizado organizacional - melhoria contínua”, apesar de não ser uma área fortalecida - mesmo no período diurno, teve mais respostas favoráveis nesse turno que no noturno (72,0% no diurno vs. 60,4% no noturno). Quanto ao “retorno da informação e comunicação sobre os erros”, também houve diferença expressiva entre os dois turnos, sen- do essa uma dimensão considerada frágil no período noturno (63,1% no diurno vs. 48,0% no noturno). A “adequação de pessoal” foi frágil em ambos os períodos analisados, no entanto mostrou-se ainda mais fragilizada durante o dia (36,3% no diurno vs. 47,7% no noturno). As comparações das demais dimensões por turno de trabalho podem ser observadas na Tabela 2.

**Tabela 2:** Frequência de respostas positivas, neutras e negativas para as doze dimensões da cultura de segurança do paciente por turno de trabalho

	Positiva	Diurno Neutra	Negativa	Positiva	Noturno Neutra	Negativa
Trabalho em equipe dentro das unidades (%)	70,1 183/261	20,3 53/261	9,6 25/261	64,8 129/199	19,1 38/199	16,1 32/199
Expectativas do supervisor/chefe e ações promotoras de segurança (%)	71,8 188/262	19,1 50/262	9,2 24/262	68,5 137/200	24,5 49/200	7,0 14/200
Aprendizado organizacional (%)	72,0 139/193	20,7 40/193	7,3 14/193	60,4 90/149	24,8 37/149	14,8 22/149
Apoio da gestão hospitalar para a segurança do paciente (%)	67,2 129/192	21,9 42/192	10,9 21/192	63,3 93/147	19,0 28/147	17,7 26/147
Percepção geral de segurança (%)	52,7 138/262	26,7 70/262	20,6 54/262	54,0 107/198	27,3 54/198	18,7 37/198
Retorno da informação e comunicação sobre os erros (%)	63,1 125/198	25,3 50/198	11,6 23/198	48,0 71/148	25,7 38/148	26,4 39/148
Abertura da comunicação (%)	43,4 86/198	36,4 72/198	20,2 40/198	44,0 66/150	32,0 48/150	24,0 36/150
Frequência da notificação de eventos de segurança (%)	77,2 152/197	11,7 23/197	11,2 22/197	60,7 91/150	15,3 23/150	24,0 36/150
Trabalho em equipe entre as unidades hospitalares (%)	47,2 120/254	34,6 88/254	18,1 46/254	40,0 78/195	35,9 70/195	24,1 47195
Adequação de pessoal (%)	36,3 94/259	29,0 75/259	34,7 90/259	47,7 94/197	22,3 44/197	29,9 59/197
Mudanças de turno e transições entre unidades/serviços (%)	41,2 105/255	32,2 82/255	26,7 68/255	44,7 88/197	28,4 56/197	26,9 53/197
Respostas não punitivas aos erros (%)	24,5 47/192	27,1 52/192	48,4 93/192	17,3 26/150	29,3 44/150	53,3 80/150

Fonte: Elaboração própria a partir dos dados compilados no estudo

## Discussão

O objetivo deste trabalho foi observar a cultura de segurança do paciente em um hospital filantrópico brasileiro. Essa medida vem recebendo interesse crescente tanto de pesquisadores quanto de profissionais de saúde por fornecer apontamentos sobre as áreas mais fortes e frágeis dentro de uma organização e que, portanto, requerem maior atenção.(26) Neste trabalho, a distribuição homogênea em diferentes setores e turnos de trabalho dos participantes incluídos pode ser considerada como representativa dos profissionais de Enfermagem da instituição, permitindo confiabilidade aos achados. Sem deixar de levar em conta a peculiaridade contextual da organização estudada, os resultados deste trabalho podem se assemelhar a diferentes cenários, possibilitando reflexões e aprendizados.

Pesquisas evidenciam que mesmo países desenvolvidos, como os Estados Unidos, apresentam falhas na cultura de segurança. Porém, constata-se que em países em desenvolvimento, como o Brasil, essas falhas podem ser ainda maiores. Em países como o nosso, as condições para uma segurança adequada podem ser menores por serem mais comuns certas precariedades nos serviços como, a escassez de equipamentos e recursos humanos, a sobrecarga de trabalho, a falta de motivação pelos profissionais relacionada à sua baixa valorização, e a elevada hierarquização entre as diferentes profissões(17).

Dentre as diferentes dimensões de cultura de segurança observadas nesse estudo, nenhuma foi considerada fortalecida, o que pode relacionar-se ao fato de a segurança do paciente ser uma temática que vem sendo abordada de maneira mais explícita em período ainda recente em nosso país. Resultados como esse também foram observados em outros estudos. O conjunto de pesquisas incluídas em revisão sistemática que abordou a cultura de segurança revelou que as culturas organizacionais hospitalares são predominantemente fracas em relação à segurança do paciente(11), sendo necessário seu fortalecimento em diferentes aspectos.

A aplicação do instrumento HSOPSC permitiu observar o ponto de vista dos profissionais quanto às diferentes dimensões relacionadas à cultura de segurança do paciente, tendo sido identificadas como áreas frágeis e que, portanto, necessitam de intervenções: a “mudança de turno e transição entre as unidades”, a “abertura da comunicação”, o “trabalho em equipe entre as unidades hospitalares”, a “adequação de pessoal”, e a “resposta não punitiva ao erro”. O instrumento utilizado, HSOPSC, traduzido em diferentes idiomas e adaptado transculturalmente em diferentes localidades, possibilitou, ainda, diferentes comparações dos resultados deste trabalho com outras pesquisas(7). Em um estudo realizado em um Hospital Universitário do Rio de Janeiro foi encontrado resultado semelhante no que diz respeito à dimensão“mudança de turno e transição entre as unidades” (17). Segundo os autores do estudo(17), a fragilidade dessa dimensão pode comprometer a assistência por refletir as perdas de informações e a fragmentação do cuidado, algo que envolve um alto risco de incidentes e que deve ser superado para que haja uma assistência segura.

Essa dimensão está muito relacionada a outra, a“abertura da comunicação”, também identificada como frágil no nosso estudo e em outro estudo nacional(17). O fato de o profissional não ter liberdade com seu superior para discutir algumas questões e não ser ouvido pode ter grande influência nesse aspecto. Por isso, é de grande importância salientar a necessidade de gestores e demais líderes criarem um canal de comunicação efetivo com os profissionais prestadores do cuidado direto ao paciente, reconhecendo as limitações e desafios cotidianos enfrentados pelos profissionais da linha de frente e também sua capacidade de contribuir frente às suas vivências e experiências.

Também relacionada à comunicação e transição do cuidado, o “trabalho em equipe entre as unidades hospitalares” foi outra dimensão identificada como fragilizada. Essa dimensão envolve a percepção dos colaboradores sobre a coordenação e cooperação entre as unidades hospitalares, com vistas a oferecer o melhor atendimento possível ao paciente. Todavia, a individualidade entre setores ainda é uma realidade e um grande desafio a ser abordado7. O pensamento voltado ao cuidado individual, e não ao coletivo e integral, cria um distanciamento e barreiras entre os setores e serviços, prejudicando o cuidado.

Em relação à “adequação de pessoal”, este estudo reforçou a necessidade de adequação do quantitativo de profissionais, uma realidade observada em diferentes cenários. Em revisão sistemática que incluiu trinta e três estudos conduzidos em diferentes países - a maioria desenvolvidos - foi identificado que a maior parte dos trabalhos apontou a “adequação de pessoal” como uma dimensão fragilizada(7), revelando que o dimensionamento adequado da equipe de Enfermagem constitui um desafio mundial, presente não somente em países em desenvolvimento. No nosso estudo o número inadequado de profissionais foi ainda mais evidente no período diurno (com apenas 36,3% das respostas positivas vs. 47,4% do período noturno). Durante o dia, a sobrecarga de trabalho pode ser ainda maior devido a outros tipos de demandas de trabalho além da prestação direta do cuidado ao paciente.

Diferentes estudos internacionais têm evidenciado o impacto das características da equipe de Enfermagem - número de pessoal e composição da equipe - na qualidade da assistência prestada(19), sendo observadas influências dessas características na segurança e mortalidade dos pacientes. Griffits et al. (2019)(20) conduziram um estudo observacional retrospectivo em um grande hospital localizado no sul da Inglaterra entre os anos de 2012 e 2015 e verificaram resultados relevantes, como o risco aumentado de morte em 3% para cada dia de internação quando o número de enfermeiros graduados estava abaixo da média esperada para a enfermaria. Diante disso, e como alternativa ao número deficiente de profissionais, cabe ao gestor da instituição dar mais autonomia ao enfermeiro compor-se de habilidades gerenciais que permitam elaborar a previsão e provisão dos recursos humanos de Enfermagem com atenção às atividades legalmente previstas(21).

A dimensão que se refere à “resposta não punitiva ao erro” também foi observada como fraca. Esse mesmo resultado também foi identificado em 70% dos estudos incluídos na revisão sistemática sobre a cultura de segurança(7), salientando o quanto a cultura de culpa está arraigada nas organizações. De certa forma, o erro ainda está associado à culpa, ao ambiente de trabalho punitivo e a uma cultura de pensar que os erros provocados pelo cuidador são resultado de descuido. Sendo assim, a criação de um elo de confiança entre os profissionais deve ser bem firmada no serviço devido à importância de se discutir o erro, e não escondê-lo, por medo de ser punido. Por isso, ao invés de uma cultura de culpa, onde erros são vistos como fracassos pessoais, deve haver uma cultura justa, onde os erros sejam encarados como oportunidades de melhorar o sistema através de estratégias de segurança do paciente voltadas para a prevenção(7)-(22).

De maneira geral, pode-se considerar que a caminhada rumo à superação desse desafio está sendo trilhada na organização, uma vez que apesar dessa fragilidade quanto à “resposta não punitiva ao erro”, a dimensão que trata da “frequência de notificação de eventos de segurança” não foi fraca de uma maneira geral (69,7% de respostas positivas na análise global; 77,2% no período diurno; 60,7% no período noturno). De maneira oposta, muitas pesquisas apontam essa dimensão como frágil(23), uma vez que a punição ao erro, relacionando-o à incompetência, pode ser uma das razões que levam o profissional a não realizar a notificação de eventos(24).

No Brasil, a notificação dos eventos é prevista pelo Programa Nacional de Segurança do Paciente, e tem a finalidade de investigar, analisar e monitorar os incidentes nos serviços.(6) Contudo, sabe-se que essa exigência por si só não é suficientemente relevante para tornar frequente o ato de notificar e para cumprir com seu principal objetivo referente ao aprendizado e mudanças de conduta. Salienta-se, contudo, diante das diferenças observadas para a “frequência de notificação de eventos de segurança” por turnos de trabalho, a importância de que essas ações ocorram de forma completa na instituição.

De forma coerente, o “aprendizado organizacional - melhoria contínua” também foi uma dimensão mais forte no hospital estudado. Essa dimensão diz respeito à capacidade de aprendizagem, que deve ser um fenômeno contínuo e ocorrer de maneira formal e informal(7). No entanto, assim como na “frequência de notificação de eventos de segurança”, também foi observada maior força para o “aprendizado organizacional” no período diurno (72,0% nesse turno vs. 60,4% no período noturno), o que pode ser um indicativo de que as iniciativas de mudança e melhoria ocorrem mais frequentemente durante o dia, alertando para o perigo de que essas ações estejam mais voltadas a demandas externas, como a busca por acreditação por exemplo, em detrimento das necessidades organizacionais próprias. A grande diferença na dimensão “retorno da informação e comunicação sobre os erros” entre os turnos de trabalho - observada como fragilizada no período noturno (48,0% de respostas positivas à noite vs. 63,1% durante o dia) - ressaltam ainda mais esse risco. Por outro lado, essa diferença pode apenas representar uma mudança paulatina, que pode acontecer em qualquer serviço.

Assim como a “frequência de notificação de eventos de segurança” e o “aprendizado - organizacional - melhoria contínua”, outras dimensões também mostraram-se mais fortes na instituição, com respostas que não estiveram acima de 75% - conforme o previsto - mas com valores acima de 65%. Entre essas estiveram, a “expectativa do supervisor e ações promotoras de segurança”, o“trabalho em equipe dentro das unidades”, e o“apoio da gestão hospitalar para a segurança do paciente”.

O “trabalho em equipe dentro das unidades” parece ser uma das dimensões com maior possibilidade de força dentro das organizações(25)-(28). Isso pode ser explicado pela necessidade de apoio mútuo e trabalho conjunto necessários à atuação da equipe de Enfermagem e ressaltados desde a graduação. Contudo, o fato de essas características estarem mais presentes apenas dentro de uma mesma equipe que atua em um setor específico, e não extrapolarem para outros setores e turnos na transição do cuidado, como evidencia nosso próprio estudo, constituem uma barreira a ser superada.

O “apoio da gestão hospitalar para a segurança do paciente” e a “expectativa do supervisor e ações promotoras de segurança” são dimensões essenciais na trajetória rumo a uma cultura de segurança fortalecida. O apoio de lideranças diversas é essencial para a disseminação de um movimento rumo a mudanças e pode funcionar como um motor que impulsiona novas práticas mais seguras e a adoção de novas rotinas estabelecidas.

Apesar de não ter sido realizada neste estudo a comparação das dimensões de cultura por fatores como: idade, profissão - nível técnico e superior, e tempo em serviço, sabe-se que essas características dos profissionais também podem influenciar na cultura de segurança ao paciente. Os profissionais incluídos nessa pesquisa tinham tempo médio de formação relativamente curto, 6 anos, o que pode favorecer uma cultura de segurança mais positiva. Os profissionais mais jovens, com menor tempo de profissão, podem ser mais flexíveis e abertos a mudanças, quando novas rotinas não representam algo assustador e diferente. Esses profissionais podem estar mais disponíveis para adquirirem um novo conhecimento e uma nova maneira de exercer seu trabalho. Ao contrário, profissionais mais velhos, que exercem o oficio por mais tempo, podem atuar de forma mecânica e serem resistentes a mudanças, o que dificulta a implementação de novas condutas. Um estudo nacional, que avaliou a cultura de segurança do paciente em centro cirúrgico, identificou maior relação com respostas positivas sobre a cultura de segurança entre os profissionais de grupos etários mais jovens(29).

Vale destacar que esta pesquisa realizou a avaliação da cultura de segurança do paciente somente com a equipe de Enfermagem, ainda que seja reconhecido que a cultura de segurança envolve a equipe multiprofissional. Isso pode ser considerado uma lacuna, ou limitação, deste trabalho. Porém, além de tratar-se de um trabalho desenvolvido por acadêmicas do curso de Enfermagem, motivadas a reconhecer esse aspecto na área, deve-se ressaltar que a equipe de Enfermagem compõe o maior número de profissionais nas instituições hospitalares, permanecendo por longo tempo ao lado do paciente, além de possuir habilidades para o planejamento, implementação e avaliação dos cuidados(25). A equipe de Enfermagem, portanto, tem uma relação direta com a segurança do paciente, sendo parte fundamental na contribuição para a disseminação dessa cultura na organização e entre os demais profissionais.

## Conclusão

A análise da cultura de segurança nesse hospital contribuiu para mais conhecimento sobre os fatores capazes de influenciá-la. Foram observadas, tanto áreas de maior força, como áreas mais fragilizadas, onde faz-se necessário o aperfeiçoamento de condutas para garantir uma assistência mais segura.

São necessárias, principalmente, mudanças de rotinas voltadas para: i) canais de comunicação mais efetivos entre os profissionais e maior abertura e capacidade de ouvir por parte das lideranças; ii) maior cooperação e coordenação entre as unidades hospitalares; iii) dimensionamento adequado da equipe de Enfermagem, principalmente no período diurno de trabalho; iv) maiores esforços na disseminação da informação de que erros não são intencionais, permeados por fatores diversos e, portanto, não individuais e passíveis de punição; e v) alerta para o risco quanto à supervalorização de demandas externas ao próprio trabalho e objetivos organizacionais.

Esses resultados podem servir como um guia rumo a mudanças mais direcionadas à própria organização estudada e a outros serviços, mas também podem ser encarados como um incentivo para que outros gestores adotem essa ferramenta de avaliação e estejam atentos às dimensões da cultura de segurança que requerem maior atenção em seus espaços de atuação.
